# Antiplatelet therapy to prevent ischemic events in giant cell arteritis: protocol for a systematic review and meta-analysis

**DOI:** 10.1186/s13643-024-02599-w

**Published:** 2024-07-08

**Authors:** Jean-Paul Makhzoum, Youssef Baati, Octavian Tanase, Arielle Mendel, Christian Pagnoux, Carolyn Ross

**Affiliations:** 1 Vasculitis Clinic, Hopital Sacre-Coeur, University of Montreal, Canadian Vasculitis Research Network, 5400 Bd Gouin O, Montreal, QC H4J1C5 Canada; 2 Hopital Sacre-Coeur, University of Montreal, 5400 Bd Gouin O, Montreal, QC H4J1C5 Canada; 3 Vasculitis and Lupus Clinic, McGill University Health Center, McGill University, Canadian Vasculitis Research Network, 1001 Bd Decarie, Montréal, QC H4A 3J1 Canada; 4 Vasculitis Clinic, Mount Sinai Hospital, University of Toronto, Canadian Vasculitis Research Network, 60 Murray St, Toronto, ON M5T3L9 Canada

**Keywords:** Giant cell arteritis, Antiplatelet therapy, Ischemic events, Visual impairment, Stroke, Myocardial infarction

## Abstract

**Background:**

Giant cell arteritis (GCA) is the most common systemic vasculitis in adults. Presenting features include new-onset headaches, constitutional symptoms, jaw claudication, polymyalgia rheumatica, and visual symptoms. Arterial inflammation with subsequent stenosis and occlusion may cause tissue ischemia, leading to blindness, strokes, and myocardial infarction. Oral antiplatelet therapy has been hypothesized to reduce GCA-related ischemic events. However, previous studies have demonstrated conflicting results regarding the efficacy of antiplatelet agents in GCA. The objective of this systematic review is to assess the safety and efficacy of antiplatelet therapy for the prevention of these events in adults with giant cell arteritis.

**Methods:**

In this systematic review, we will include randomized controlled trials (RTCs), quasi-randomized trials, non-randomized intervention studies, cohort studies, and case–control studies on patients with new-onset or relapsing GCA. The intervention of interest will be pre-existing use or initiation of an oral antiplatelet medication (aspirin, clopidogrel, prasugrel, or ticagrelor) at GCA onset or relapse. The comparator of interest will be the absence of antiplatelet therapy. Endpoints will be evaluated after 6 and 12 months of follow-up. The primary outcome will be GCA-related ischemic events, including permanent blindness, stroke, myocardial infarction, and ischemic event-related deaths. Adverse events such as major bleeding and death caused by a bleeding event will be assessed.

**Discussion:**

GCA-related ischemic events are catastrophic, sudden, often irreversible, and lead to significant morbidity. Antiplatelet agents are affordable, accessible, and could be effective for the prevention of these events. Nevertheless, the potential benefits of platelet aggregation inhibition must be weighed against their associated risk of bleeding. Assessing the efficacy and safety of antiplatelet therapy in GCA is therefore clinically important.

**Systematic review registration:**

Our systematic review protocol was registered with the International Prospective Register of Systematic Reviews (PROSPERO, registration number CRD42023441574.

**Supplementary Information:**

The online version contains supplementary material available at 10.1186/s13643-024-02599-w.

## Background

Giant cell arteritis (GCA) is the most common systemic vasculitis in adults [1]. GCA causes inflammation of large arteries and has an incidence of 15–20 cases per 100,000 people in those over 50 years of age [2]. Common features include new-onset headaches, constitutional symptoms, jaw claudication, visual symptoms, and polymyalgia rheumatica [3–6].

GCA is treated with oral glucocorticoids which are usually tapered over a period of 12 to 18 months [1]. Depending on the clinical phenotype, additional immunosuppressive therapy is sometimes required [7, 8]. Symptomatic relapses are common and occur in 40–80% of patients during glucocorticoid tapering or discontinuation [9, 10].

Complications of GCA can be severe. Arterial inflammation with subsequent stenosis and occlusion may cause GCA-related ischemic events. These include permanent blindness in 15–20% of patients, strokes in 3–8% of patients, and myocardial infarction in 2–4% of patients [11–13]. Moreover, GCA often affects patients over 70 years of age, and older age is recognized as a non-modifiable risk factor for ischemic events [14]. GCA-related ischemic events, which are often irreversible, typically occur within 1 year following disease onset or relapse [10], and may happen despite appropriate glucocorticoid or immunosuppressive therapy [15].

Pathophysiology of ischemic events in GCA is complex and incompletely understood. Arterial inflammation, myointimal thickening, and endothelial dysfunction can trigger platelet activation and aggregation [16]. This potentially leads to the narrowing and/or thrombotic occlusion of the inflamed arteries, leading to organ ischemia. Potential benefits of oral adjunctive antiplatelet therapy have been hypothesized to reduce ischemic events since they inhibit platelet aggregation and thrombus formation [17]. Furthermore, antiplatelet agents may have immune-mediated effects by suppressing interferon-gamma transcription in arterial tissue resident cells [18]. The addition of antiplatelet therapy to oral glucocorticoids may therefore provide a synergistic effect in the treatment of GCA [19].

Assessing the efficacy of antiplatelet therapy to prevent these events is critically important. Moreover, these agents are affordable and accessible. Nevertheless, the potential benefits of platelet aggregation inhibition must be weighed against their associated risk of bleeding [20]. Commercially available and commonly prescribed antiplatelet medications include aspirin, clopidogrel, ticagrelor, and prasugrel.

Previous small and mostly retrospective studies have demonstrated conflicting results regarding the efficacy of antiplatelet agents in reducing GCA-related permanent vision loss [1, 11, 17, 18].

A Cochrane systematic review attempted to assess the safety and effectiveness of low‐dose aspirin, as an adjunctive therapy, in the treatment of GCA [21]. However, observational studies were excluded, and only randomized controlled trials (RCTs) directly comparing outcomes of GCA with and without concurrent adjunctive use of low‐dose aspirin were eligible for inclusion, and therefore no studies met the inclusion criteria. Furthermore, the review was conducted before the results of major therapeutic clinical trials in GCA were available.

The objective of this systematic review and meta-analysis is to assess the safety and efficacy of antiplatelet therapy for the prevention of ischemic events in adults with GCA. For efficacy assessment, we will focus on the incidence of specific GCA-related ischemic events including permanent blindness, stroke, myocardial infarction, and ischemic event-related deaths. The safety outcomes will be clinically significant adverse events such as major bleeding events and death caused by a bleeding event.

## Methods/design

This systematic review protocol is reported according to the guidelines of the Preferred Reporting Items for Systematic Reviews and Meta-Analyses Protocols (PRISMA-P) [22].

### Research questions

The aim of this systematic review is to evaluate the safety and efficacy of antiplatelet therapy, in addition to standard of care, compared to no antiplatelet therapy, in adult patients with new-onset or relapsing GCA. The proposed systematic review will aim to answer the following questions:

i) In adult patients with new-onset or relapsing GCA, does antiplatelet therapy reduce GCA-related ischemic complications (permanent blindness, stroke, myocardial infarction, and ischemic event-related deaths)?
ii) In adult patients with new-onset or relapsing GCA, does antiplatelet therapy increase the risk of major bleeding events?

### Eligibility criteria

Eligibility criteria for studies based on patient population, intervention or exposure, comparator, and methods are presented in Table 1. We will include studies irrespective of the reported outcomes. We will include randomized controlled trials (RTCs), quasi-randomized trials, and non-randomized intervention studies (with a planned intervention and control without randomization) [23]. For observational studies (where patients are routinely treated and observed without any intervention by the investigators), we will include cohort studies and case–control studies. We will exclude cross-sectional, case series, and case reports.

**Table 1:** Eligibility criteria for studies

**Population**	*Inclusion criteria* • Adult patients (≥ 18 years), with new-onset or relapsing GCA based on one of the official ACR classification criteria (version 1990 or version 2022). *
	*Exclusion criteria* • Patients with systemic vasculitides other than GCA
**Intervention/exposure**	*Inclusion criteria* • Administration of an oral antiplatelet medication in addition to GCA standard of care • Accepted antiplatelet medications: aspirin (≥ 80 mg daily), clopidogrel (75 mg daily), ticagrelor (90 mg twice a day), or prasugrel (10 mg daily) • Accepted timing of antiplatelet initiation: o Within 8 weeks of GCA onset or relapse o Already administered at the time of GCA onset or relapse
	*Exclusion criteria* • Antiplatelet medication initiated in a patient with inactive GCA • Use of oral, intravenous, or subcutaneous anticoagulants
**Comparator**	*Inclusion criteria* • Absence of adjunctive antiplatelet therapy
	*Exclusion criteria* • Use of oral, intravenous, or subcutaneous anticoagulants
**Method/design**	*Inclusion criteria* • RTCs, quasi-randomized trials, non-randomized intervention studies, cohort studies and case–control studies • Study duration of at least 6 months
	*Exclusion criteria* • Cross-sectional, case series, and case reports

*GCA*, giant cell arteritis; *ACR*, American College of Rheumatology; *RCT*, randomized controlled trial

^*^New-onset GCA is defined as a diagnosis of active GCA in a participant with no previous history of GCA. A relapsing GCA is defined as a diagnosis of active GCA in a patient with a history of GCA in remission (asymptomatic)

A study duration of at least 6 months will be required for study inclusion. Endpoints will be evaluated at 6 and 12 months.

We will include trials irrespective of the language of publication or format in which they were reported. If we identify studies with unpublished data, they will be considered for inclusion. There will be no restriction by type of setting (academic hospital, community hospital, inpatient, or outpatient).

### Outcomes

The primary composite efficacy outcome is the incidence (proportion) of GCA-related ischemic events occurring during the follow-up period, which include ischemic strokes, permanent blindness, myocardial infarction, or ischemic event-related deaths. The definition of ischemic events is based on the official American Heart Association (AHA) and American Stroke Association (ASA) consensus. Ischemic stroke is defined as an episode of neurological dysfunction caused by focal cerebral, spinal, or retinal infarction. Permanent blindness is a permanent loss of sight, whether it be a full or partial loss. Myocardial infarction is defined as an episode of myocardial injury (elevated cardiac troponin values at least above the 99th percentile upper reference limit) with clinical evidence of at least one acute myocardial ischemia manifestation (symptoms of cardiac ischemia, new ischemic ECG changes, new pathological Q waves, compatible cardiac imaging, or coronary thrombus on angiography).

The main secondary efficacy outcome is the incidence (proportion) of ischemic strokes, permanent blindness, myocardial infarction, and death, measured separately and occurring during the follow-up period.

The main safety outcome is the incidence (proportion) of major bleeding events. A major bleeding event is defined based on the classification of the International Society of Thrombosis and Hemostasis as either the following: fatal bleeding, and/or symptomatic bleeding in a critical area or organ, such as intracranial, intraspinal, intraocular, retroperitoneal, intra-articular or pericardial, or intramuscular with compartment syndrome, and/or bleeding causing a fall in hemoglobin levels of 20 g/L or more, and/or leading to a transfusion.

The main secondary safety outcome is the incidence (proportion) of death due to a bleeding event.

### Information sources

Available published studies and unpublished gray literature will be searched. We will search the following electronic bibliographic databases: MEDLINE (Ovid interface, January 1946–onwards), Cochrane Central Register of Controlled Trials in the Cochrane Library (CENTRAL), and EMBASE (Ovid interface, January 1947–onwards). The following study registries will be searched: metaRegister of controlled trials (mRCT) and ClinicalTrials.gov.

Available online conference papers, abstracts, and presentations from the American College of Rheumatology annual meeting (ACR, from 2012–onwards) and the European League Against Rheumatism annual meeting (EULAR, from 2001–onwards) will be searched.

References will be screened to make a list of experts in the field. A citation index search of these experts will be performed on the “Web of Science” platform and “researchgate.net.” Finally, a manual bibliography search of retrieved records will be performed to find additional references.

We will search for retraction or errata statements that were published for every study we include.

### Search strategy

Literature search strategies were developed using medical subject headings (MeSH) and text words related to GCA and antiplatelet therapy. There will be no language restriction for the search. A publication date filter from August 1st, 1990–onwards will be used because official classification criteria for GCA were released in August 1990. This will allow correct identification of the population of interest.

**Fig. 1:**
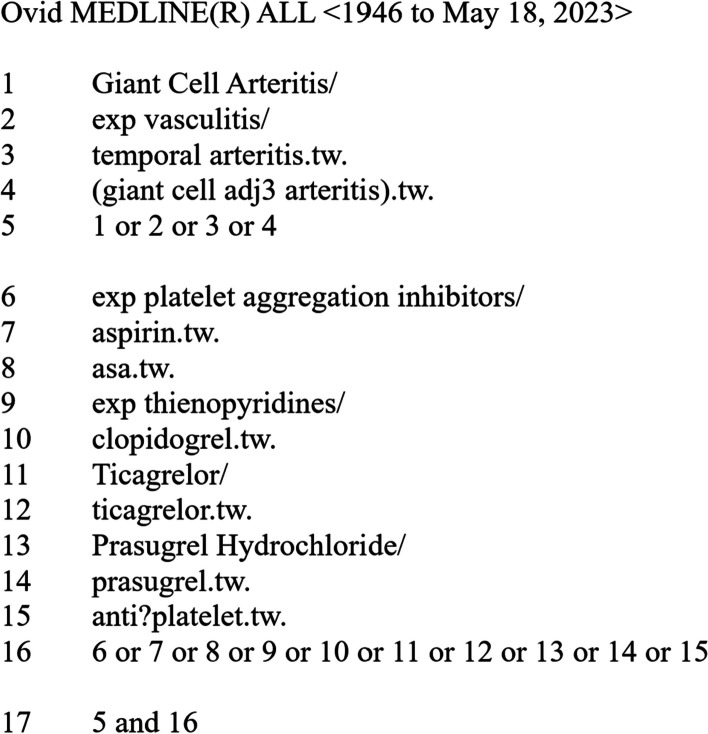
MEDLINE (OVID) search strategy

The search strategy was elaborated by the corresponding author and was peer-reviewed by all authors. A transcript of the MEDLINE search strategy is provided (Fig. 1). Search strategies for other information sources are provided in the data supplement (Additional file 1: Figs. S1 to S5). We will provide the actual date when each search was performed during the review stage.

### Study records management, selection, and collection

Covidence (covidence.org) will be used to upload literature search results, manage study records, select studies, and for data extraction. Two co-authors (OT, YB) will work independently for the initial screening (title and abstract), selection of studies, and data extraction. The two reviewers will have a practice-run with 10 records as a calibration exercise before beginning the official review process.

Duplicate records will be counted and removed using Covidence. Multiple reports of the same study will be identified by juxtaposing author names, location, setting, and date of the study. Multiple reports of the same study will not be discarded; they will be collated under one study identification. However, one record will be selected as the source (main report) of the study with a justification provided.

Any disagreement between the two reviewers will be resolved through discussion. A third reviewer (CR) will be consulted if the disagreement persists. If required, additional information from the study authors will be requested to resolve the remaining questions on eligibility.

### Data items

Extracted data will include identification information (study id, record id), reason for excluding studies, characteristics of the study (author, year, country, design, duration, funding, conflict of interest), characteristics of participants (age, sex, ethnicity, disease subtype, baseline characteristics), details of the intervention (timing of initiation, type of antiplatelet medication, dose, other GCA therapy and immunosuppression), details of the comparator, outcomes (definitions, method of aggregation, measures of association, timing), and results (number of participants, exclusion, losses at follow-up, summary results, subgroup results). Furthermore, key conclusions, comments, and references will be collected.

Missing data will be recorded as such. Study authors will be contacted to retrieve missing data or resolve any uncertainties. Communications with study authors will be performed by email. A maximum of 3 attempts to reach the authors will be made in case of no response, with each attempt every 14 days.

### Risk of *bias* of individual studies

For randomized studies, we will use version 2 of the Cochrane Collaboration tool for assessing the risk of bias (RoB 2) [24]. For non-randomized intervention studies, the Risk Of Bias In Non-randomized Studies–of Interventions (ROBINS-I) tool will be used [25]. For observational studies, the Risk Of Bias In Non-randomized Studies–of Exposure (ROBINS-E) tool will be used [26].

The risk of bias will be assessed independently by two reviewers (OT, YB). Disagreements will be resolved by discussion, and if required, with arbitration from a third reviewer (JPM). Using the software RevMan web, we will summarize our findings and present them in a risk of bias table and figure.

### Data synthesis

If included studies are sufficiently homogeneous in terms of participants (similar proportions of new-onset or relapsing GCA), interventions (antiplatelet type), comparator, and study design (duration of follow-up), we will perform meta-analyses using a random-effects model. Our data will be binary: proportions of GCA-related ischemic complications, major bleeding events, and deaths. We will use a Mantel–Haenszel method for quantitative synthesis, with odds ratio (OR) as a measure of association with 95% confidence intervals.

A sensitivity analysis will be performed to evaluate the impact of studies with significant missing data (when there is ≥ 10% missing data for any outcome).

Statistical heterogeneity will be tested using chi^2^ test (significance level of 0.1) and *I*^2^ statistic. If there is a high level of statistical heterogeneity (*p* < 0.1 or *I*^2^ > 50%), we will analyze clinical heterogeneity by documenting the variability in participants, interventions, and outcomes in the included trials. We will also carefully analyze and compare study designs and settings to assess methodological heterogeneity.

Furthermore, we will perform the following subgroup analyses to better understand the source of heterogeneity: (1) subgroup based on disease subtype (new onset vs. relapsing), (2) based on the timing of antiplatelet medication (prior to GCA vs. at GCA onset or relapse), (3) based on GCA therapy received (glucocorticoids alone vs. glucocorticoids with immunosuppression).

We will also perform the following sensitivity analyses: (1) exclusion of non-randomized studies, (2) exclusion of studies with a high risk of bias.

If quantitative analysis is not appropriate, a systematic narrative synthesis will be provided.

### *Meta*-biases assessment

Publication bias will be evaluated with a funnel plot if at least 10 studies are included in the meta-analysis. We will use Egger’s test to assess potential publication bias via funnel plot asymmetry. For each included study, outcome reporting bias will be investigated by comparing reported outcomes against planned outcome measures “a priori” in the study protocol or trial registry.

The risk of bias due to selective outcome reporting will be considered low if (1) the study protocol was published before the availability of study results, and (2) every “a priori” outcome in the protocol is reported in the study record (or if justification was provided for not reporting an outcome).

If no study protocol is found, we will use the Outcome Reporting Bias in Trials (ORBIT) classification system to evaluate the risk of selective outcome reporting [27].

A sensitivity analysis to assess the impact of selective outcome reporting will be conducted if at least one study presents a high risk of bias due to selective outcome reporting.

### Confidence in cumulative evidence

Two reviewers (JPM, CR) will independently evaluate the quality of evidence for all outcomes using the Grading of Recommendations, Assessment, Development, and Evaluation working group methodology (GRADE) [28]. Conclusions on GRADE for each outcome will be displayed in a “summary of findings” table. An overall GRADE will be given to the body of all outcomes.

## Discussion

This systematic review will synthesize the available literature on the efficacy of antiplatelet therapy in GCA, with emphasis on a range of clinically important ischemic complications, including permanent visual loss, myocardial infarction, and strokes. These ischemic events are infrequently described in individual studies, with insufficient power to draw conclusions.

The findings of this review are anticipated to assist advocacy groups and task teams to develop practice guidelines, which will optimize the management in GCA and hopefully assist in preventing ischemic complications.

This systematic review protocol has several strengths. This review will be conducted using an established methodology, with an advanced search strategy that includes grey literature. The included individual studies will be assessed thoroughly based on each risk of bias domain using the correct tool for the study type. By including non-randomized intervention studies and observational studies, we will increase the likelihood of finding eligible studies, as opposed to previous attempts to study this important research question. Nevertheless, we anticipate some difficulties in conducting this review. Ischemic and bleeding events in GCA studies are reported with variable consistency. The review team will mitigate this by making a significant effort towards contacting study authors as necessary to obtain the required outcome information.

The results of this systematic review will be reported in a peer-reviewed journal. Any amendments made to this protocol during the review will be reported in PROSPERO and in the final manuscript.

### Supplementary Information

Additional file 1: Fig. S1. Cochrane Central Register of Controlled Trials in the Cochrane Library (CENTRAL) Search Strategy. Fig. S2. Embase Search Strategy. Fig. S3. Study Registries Search Strategy. Fig. S4. Conference Papers Search Strategy. Fig. S5. Citation Tool Search Strategy.

## Data Availability

Not applicable.

## Abbreviations

ACR: American College of Rheumatology
AHA: American Heart Association
ASA: American Stroke Association
CENTRAL: Cochrane Central Register of Controlled Trials
ECG: Electrocardiogram
EULAR: European League Against Rheumatism
GCA: Giant cell arteritis
GRADE: Grading of Recommendations, Assessment, Development, and Evaluations
MeSH: Medical Subject Headings
mRCT: MetaRegister of Controlled Trials
OR: Odds ratio
ORBIT: Outcome Reporting Bias in Trials
PROSPERO: International Prospective Register of Systematic Reviews
RCT: Randomized controlled trial
RoB: Risk of bias
ROBINS-I: Risk of Bias in Non-randomized Studies–of Interventions
ROBINS-E: Risk of Bias in Non-randomized Studies–of Exposure
