# Supplementary material for: Antiplatelet therapy to prevent ischemic events in giant cell arteritis: protocol for a systematic review and meta-analysis
Source: Syst Rev. 2024 Jul 8;13:173. doi: 10.1186/s13643-024-02599-w (PMC11229199; doi:10.1186/s13643-024-02599-w)
Supplement: Supplementary file 1 — Additional file 1: Fig. S1. Cochrane Central Register of Controlled Trials in the Cochrane Library (CENTRAL) Search Strategy. Fig. S2. Embase Search Strategy. Fig. S3. Study Registries Search Strategy. Fig. S4. Conference Papers Search Strategy. Fig. S5. Citation Tool Search Strategy. [file 13643_2024_2599_MOESM1_ESM.docx]

**Antiplatelet Therapy to Prevent Ischemic Events in Giant Cell Arteritis: protocol for a systematic review and meta-analysis**

Jean-Paul Makhzoum, MD., Youssef Baati, MD., Octavian Tanase, MD.,
Arielle Mendel, MD., MSc., Christian Pagnoux, MD., MPH., Carolyn Ross, MD., PharmD.

**Data supplement**

**Figure S1 -** Cochrane Central Register of Controlled Trials in the Cochrane Library (CENTRAL) Search Strategy

#1 Giant Cell Arteritis

#2 giant near/2 cell near/2 arteritis

#3 (temporal or cranial) near/2 (arteritis)

#4 GCA

#5 #1 or #2 or #3 or #4

#6 aspirin or ASA

#7 acetylsalicylic near/2 acid

#8 clopidogrel

#9 Fibrinolytic Agents

#10 #6 or #7 or #8 or #9

#11 #5 and #10

**Figure S2 -** Embase Search Strategy

Ovid Embase <1947 to 1973 and 1974 to 2023 June 16>

1 giant cell arteritis/

2 (giant adj2 cell adj2 arteritis).tw.

3 ((temporal or cranial) adj2 arteritis).tw.

4 GCA.tw.

5 temporal arteritis/

6 aortitis/

7 giant cell aortitis/

8 1 or 2 or 3 or 4 or 5 or 6 or 7

9 exp antithrombocytic agent/

10 exp acetylsalicylic acid/

11 exp clopidogrel/

12 (acetylsalicylic adj2 acid).tw.

13 (aspirin or ASA).tw.

14 9 or 10 or 11 or 12 or 13

15 8 and 14

**Figure S3 -** Study Registries Search Strategy

ClinicalTrials.gov

Giant Cell Arteritis

### MetaRegister of Controlled Trials search strategy

Giant Cell Arteritis

**Figure S4 -** Conference Papers Search Strategy

American College of Rheumatology (2012-onwards)

[https://www.rheumatology.org/Learning-Center/Publications-Communications/](https://www.rheumatology.org/Learning-Center/Publications-Communications/Abstract-Archives)

Giant Cell Arteritis

European League Against Rheumatism (2001-onwards)

[http://scientific.sparx-ip.net/archiveeular/](http://scientific.sparx-ip.net/archiveeular/?c=s&view=1&searchfor=giant+cell+arteritis)

Giant Cell Arteritis

**Figure S5 -** Citation Tool Search Strategy

Web Of Science

[https://www.webofscience.com/](https://www.webofscience.com/wos/woscc/summary/b1567d34-6d80-4e2e-8040-89019971d9d7-7d2b34d7/relevance/1)

Giant Cell Arteritis AND Ischemic

Research Gate

<https://www.researchgate.net/>

Giant Cell Arteritis AND Ischemic
